# Intraspecific Differentiation of *Styrax japonicus* (Styracaceae) as Revealed by Comparative Chloroplast and Evolutionary Analyses

**DOI:** 10.3390/genes15070940

**Published:** 2024-07-18

**Authors:** Hao-Zhi Zheng, Wei Dai, Meng-Han Xu, Yu-Ye Lin, Xing-Li Zhu, Hui Long, Li-Li Tong, Xiao-Gang Xu

**Affiliations:** 1 Co-Innovation Center for Sustainable Forestry in Southern China, College of Life Science, Nanjing Forestry University, Nanjing 210037, China; zhenghaozhi@njfu.edu.cn (H.-Z.Z.); weidai@njfu.edu.cn (W.D.); xumenghan2022@163.com (M.-H.X.); 13528132970@163.com (Y.-Y.L.); 15287963520@163.com (X.-L.Z.); longhui990313@163.com (H.L.); 2 State Environmental Protection Scientific Observation and Research Station for Ecology and Environment of Wuyi Mountains, Nanjing 210037, China; 3 School of Horticulture & Landscape Architecture, Jinling Institute of Technology, Nanjing 210038, China; tonglili@jit.edu.cn

**Keywords:** *Styrax japonicus*, chloroplast, adaptive evolution, morphological variation, selection pressure

## Abstract

*Styrax japonicus* is a medicinal and ornamental shrub belonging to the Styracaceae family. To explore the diversity and characteristics of the chloroplast genome of *S. japonicus*, we conducted sequencing and comparison of the chloroplast genomes of four naturally distributed *S. japonicus*. The results demonstrated that the four chloroplast genomes (157,914–157,962 bp) exhibited a typical quadripartite structure consisting of a large single copy (LSC) region, a small single copy (SSC) region, and a pair of reverse repeats (IRa and IRb), and the structure was highly conserved. DNA polymorphism analysis revealed that three coding genes (*infA*, *psbK*, and *rpl33*) and five intergene regions (*petA*-*psbJ*, *trnC*-*petN*, *trnD*-*trnY*, *trnE*-*trnT*, and *trnY*-*trnE*) were identified as mutation hotspots. These genetic fragments have the potential to be utilized as DNA barcodes for future identification purposes. When comparing the boundary genes, a small contraction was observed in the IR region of four *S. japonicus*. Selection pressure analysis indicated positive selection for *ycf1* and *ndhD*. These findings collectively suggest the adaptive evolution of *S. japonicus*. The phylogenetic structure revealed conflicting relationships among several *S. japonicus*, indicating divergent evolutionary paths within this species. Our study concludes by uncovering the genetic traits of the chloroplast genome in the differentiation of *S. japonicus* variety, offering fresh perspectives on the evolutionary lineage of this species.

## 1. Introduction

Chloroplasts in higher plants play a crucial role in the biosynthesis of amino acids, fatty acids, vitamins, and pigments [1], as well as being actively involved in photosynthesis [2]. The chloroplast genome structure is characterized by a circular quadripartite organization consisting of two inverted repeat regions (IRs), a small single copy (SSC) region, and a large single copy (LSC) region that are highly conserved [3]. Furthermore, chloroplast genes demonstrate a relatively high level of conservation and play a crucial role in fundamental biological processes such as photosynthesis, transcription, and translation [4]. The size of the chloroplast genome varies among different species, typically ranging from 120 to 160 kb, and contains two sets of four ribosomal RNA genes and 30 tRNA genes [5,6]. The majority of angiosperm species rely on matrilineal inheritance to ensure the stability of species evolution. Simultaneously, genetic mutation events provide valuable information for evolutionary studies, population classification, and species identification [7,8,9,10]. Consequently, chloroplast genes present an ideal research subject for investigating species evolution [11].

The diversity of chloroplast genomes offers valuable resources for uncovering phylogenetic relationships across various dimensions [12,13,14]. Furthermore, due to the maternal inheritance of chloroplasts in most angiosperms [15], variations in their association with nuclear phylogeny could offer valuable insights into the processes of speciation [16,17]. The modification of gene content within the chloroplast genome may contribute to the adaptation of species to specific habitats and living strategies [18,19]. Changes in the environment and habitat can impose selective genetic pressure, influencing the genes involved. Genes undergoing positive selection typically confer advantages in terms of individual fitness and reproductive capability [20]. Therefore, the investigation of gene selection pressure and adaptive evolution has emerged as a prominent area of focus in molecular research, providing a solid groundwork for the study of germplasm resources.

*S. japonicus* Siebold & Zucc is a member of the Styracaceae family, which is a low-branched landscape tree with aromatic flowers possessing significant ornamental and medicinal properties [21,22,23]. *S. japonicus* has been utilized in the treatment of various conditions, including cough, bronchitis, and laryngitis [24,25] and is also employed in traditional Chinese herbal medicine for relieving toothaches and sore throats [26]. Moreover, some scholars suggest that the distinctive properties of its nectar make it a potential additive in honey processing [27]. It exhibits wide distribution across eastern Asia, including Korea, Japan, and southern China [28]. Due to its wide distribution and morphological characteristics, there has been some confusion in the taxonomy of *S. japonicus*, making it one of the most heterogeneous species in *Styrax* [29,30,31]. However, there is limited research on *S. japonicus* variety; Ju et al. [32] conducted an analysis of the mechanism of calyx color variation in two *S. japonicus* varieties using integrated metabolomics and transcriptomics. The extent of genomic diversity in chloroplasts remains entirely unexplored.

Although the nuclear genome predominantly dictates the morphological and physiological traits of plants, the chloroplast genome indirectly influences these attributes to a notable extent. For instance, Lee and colleagues discovered variations in the chloroplast genome of *Cynanchum wilfordii*, proposing a potential link between these genetic differences and the species’ calyx variations [33]. Similarly, Tian et al., in their investigation of *Lindera obtusiloba*, highlighted variations in chloroplast genome size and intraspecific diversity, which might be connected to the plant’s adaptive responses to environmental pressures. Specifically, genes under positive selection could drive morphological adaptations in plants facing environmental stress [34]. Sarwar et al. conducted a comprehensive analysis of *Olea europaea* cultivars, revealing significant disparities in plant height, blade size and shape, phyllotaxy, venation, and chlorophyll content among varieties from different origins. These variations might originate from the diversity within the chloroplast genome [35]. In essence, the chloroplast genome’s diversity significantly shapes plant morphology, with these morphological traits not only mirroring genetic variability but also potentially indicating adaptability to specific environmental conditions. Moreover, the interplay between the chloroplast and nuclear genomes could be crucial in plant speciation and evolutionary processes. Understanding these interactions offers valuable insights into the complex genetic architecture underlying plant diversity and adaptation.

To explore the diversity and evolution of the chloroplast genome of *S. japonicus*, we collected three samples with typical leaf variation and one wild-type sample with standard morphology from the natural population of *S. japonicus* in Shandong, China, and conducted chloroplast genome sequencing. The objective is to analyze the evolutionary mechanisms, intraspecific differentiation, and chloroplast genome diversity of *S. japonicus* from the perspective of the chloroplast genome. Specifically, (1) comparative analysis of chloroplast genome variations in *S. japonicus*; (2) examination of wild type and genetic variants associated with adaptive evolution; (3) reconstruction and comparison of phylogenetic relationships.

## 2. Result

### 2.1. Chloroplast Genome Characterization and Structure

**Figure A1:**
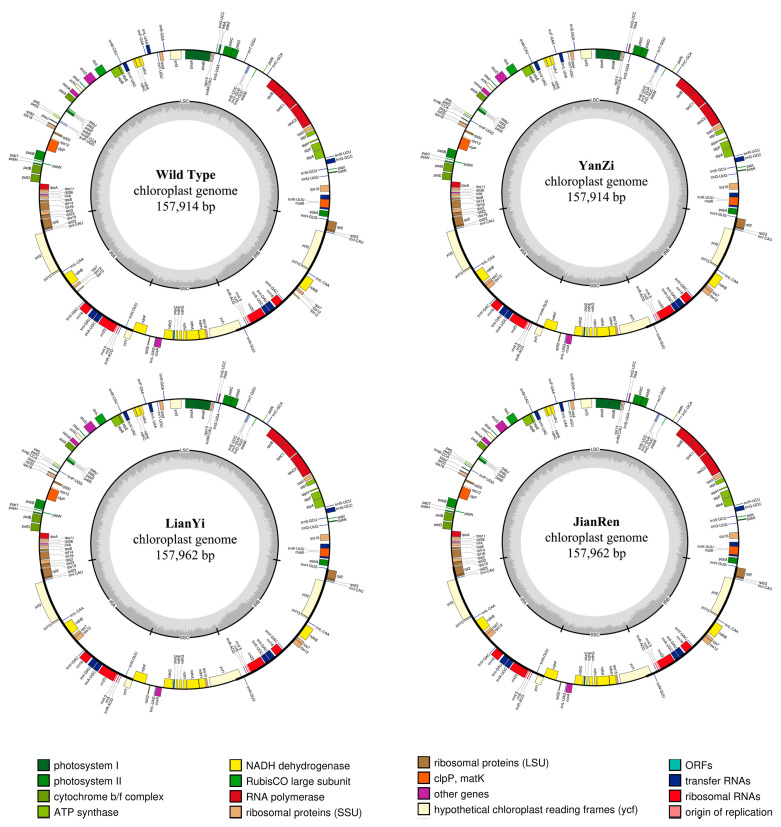
Gene map of the four *S. japonicus* chloroplast genomes.

After sequencing, the chloroplast genomes of the four samples (157,914–157,962 bp, 36.96–36.98% GC content) displayed a typical quadripartite structure, comprising of the LSC region (87,549–87,637 bp, 34.79–34.82% GC content), SSC region (18,245–18,269 bp, 30.26–30.28% GC content), and a pair of IR regions (26,040–26,048 bp, 42.94–42.95% GC content) (Appendix B Figure A1 and Table A1). Each of the three chloroplasts contains a total of 133 genes, including 87 protein-coding genes, 37 transfer RNA (tRNA) genes, 8 ribosomal RNA (rRNA) genes, and a pseudogene (*ycf1*). All genes can be categorized into three groups: (1) photosynthesis, (2) self-replication, and (3) others (Appendix B, Table A2).

### 2.2. Comparative Analysis

**Figure 1:**
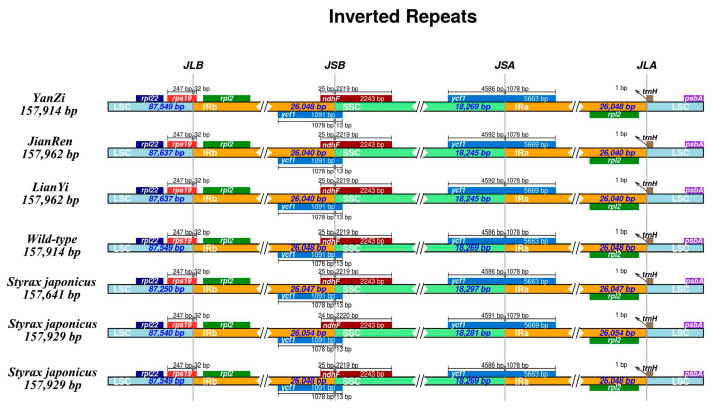
Comparison of chloroplast genome structure in four *S. japonicus* samples. IR (inverted repeat), LSC (large single copy), and SSC (small single copy) regions and border genes are indicated. Note: JLA: junction between LSC and IRa; JLB: junction between LSC and IRb; JSA: junction between SSC and IRa; JSB: junction between SSC and IRb.

**Figure 2:**
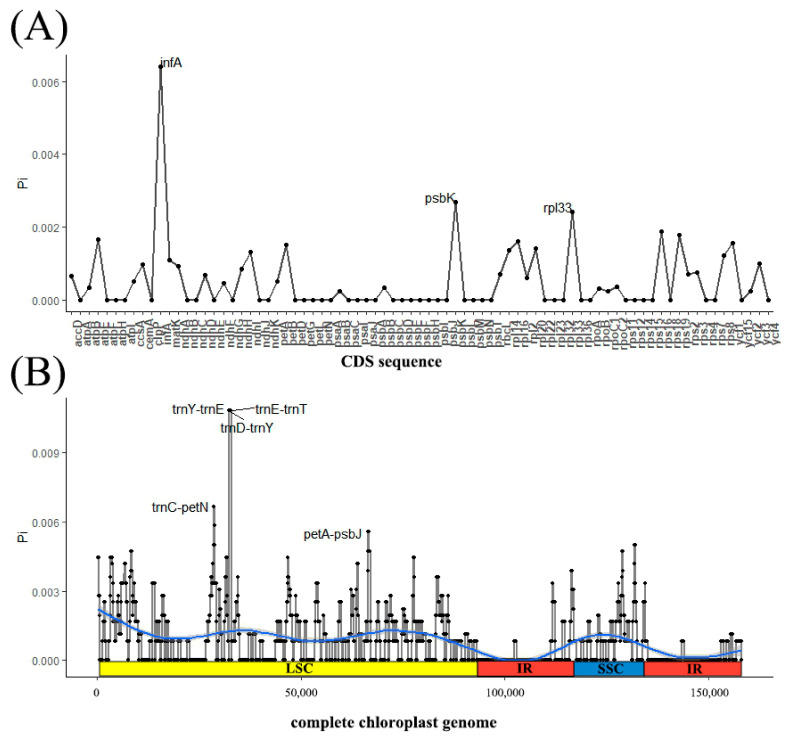
Nucleotide diversity of chloroplast genomes in *S. japonicus*. (**A**): Pi in CDS; (**B**): chloroplast genome Pi value. Note: window length: 600 bp; step length: 50 bp; *x* axis: position of the midpoint of each window; *y* axis: Pi of each window. The blue line represents the trajectory of the value of Pi.

**Figure A2:**
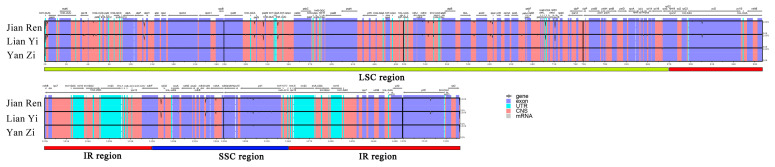
Variation level of three varieties of chloroplast genome sequences. The *y*-axis indicates the level of variation (between 50 and 100%), and the *x*-axis represents the coordinate in the chloroplast genome.

Based on the previously published *S. japonicus* chloroplast genome from NCBI (Accession number: MT178456; MZ285743; NC047429), the results indicate that four samples exhibit consistency with other *S. japonicus* boundary genes. However, there were varying degrees of expansion or contraction in the IR region (Figure 1), although such variations were minimal, with only a few bp. The chloroplast genome sequences of three varieties were analyzed using the reference chloroplast genome of wild type in mVISTA (Appendix B Figure A2), revealing a significant level of similarity and conservation among the chloroplast genomes within the three varieties. Importantly, coding regions exhibited higher levels of conservation compared to non-coding regions, while the IR regions demonstrated lower variability than the LSC and SSC regions. Through the analysis of the CDS sequence and complete chloroplast genome nucleotide polymorphisms, it was found that *infA*, *psbK*, and *rpl33* exhibited high Pi values (Figure 2A). Furthermore, the intergenic regions *petA*-*psbJ*, *trnC*-*petN*, *trnD*-*trnY*, *trnE*-*trnT*, and *trnY*-*trnE* also exhibited elevated Pi values (Figure 2B).

### 2.3. Repetitive and Codon Usage Bias Analysis

**Figure 3:**
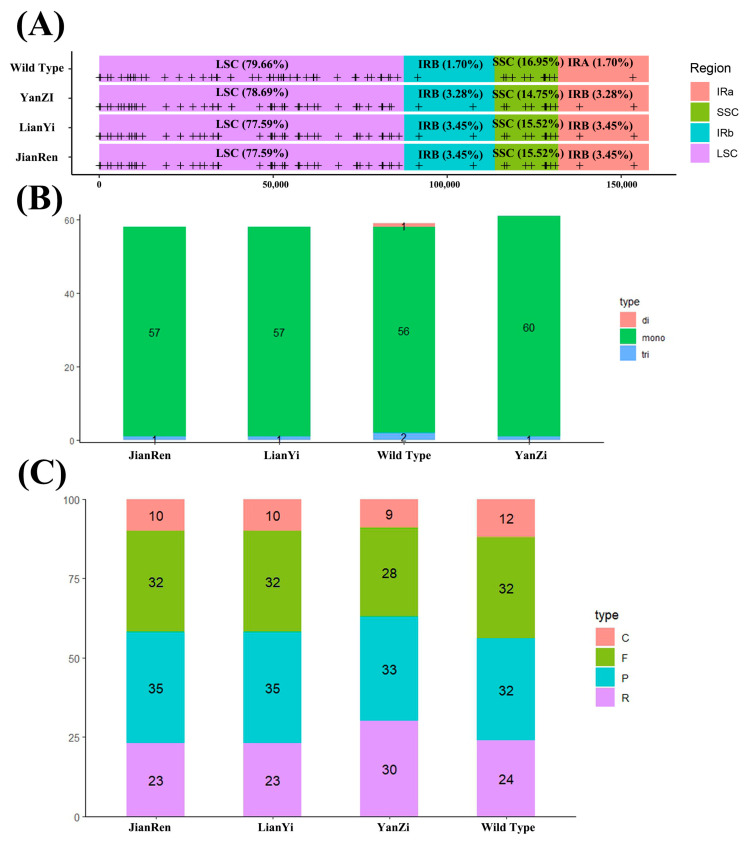
Analysis of SSR sites and repetitive sequences in five chloroplast genomes. (**A**): Distribution of SSRs in the four *S. japonicus* samples; (**B**): Number of different SSRs loci types; (**C**): number of different repeat types. Note: In (**A**), symbol (+) represented the position of SSRs and the proportion of text displayed; in (**C**), C: complementary repeats, F: forward repeats, P: palindromic repeats, R: reverse repeats.

Through SSR analysis, the four chloroplast genomes were found to contain a total of 58–61 SSRs, with the majority (77.59–79.66%) located in the LSC region. The IR regions contained between 3.39 and 6.90% of SSR loci, while the SSC region included between 14.75 and 16.95% (Figure 3A). In contrast, only WT exhibited dinucleotide repeat units (Figure 3B). Furthermore, the majority of repeats in all four species consist of mononucleotide repeats (Figure 3C).

The examination of chloroplast genomes demonstrated that the GC and GC3s composition within the codons was consistently lower than 0.5, suggesting a predilection towards A/T bases and A/T-ending codons in three chloroplast genomes.

### 2.4. RSCU Analysis and Selection in Evolution

**Figure 4:**
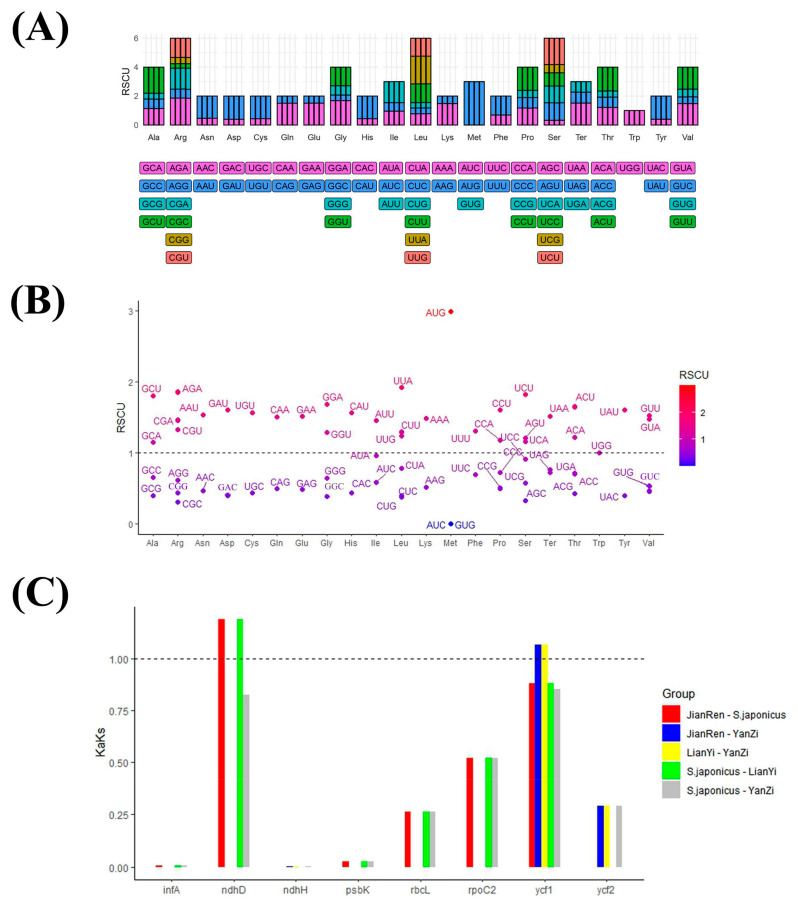
Synonymous codon usage and selective pressures in the evolution. (**A**): Codon content of 20 amino acids and stop codons in all protein-coding genes of four *S. japonicus* chloroplast genome; (**B**): Distribution of codon preference in *S. japonicus*; (**C**): Ka/Ks values of protein-coding genes of the five comparative combinations. Note: in (**A**), the top panel shows the RSCU for the corresponding amino acids, and the colored blocks, which are shown below, represent different codons. In (**C**), Ka: nonsynonymous; Ks: synonymous.

The relative synonymous codon usage (RSCU) values showed similarity across the four genomes (Figure 4A). A total of 33 codons exhibited an RSCU value greater than 1 (Figure 4B), with only two of these codons ending in G (AUG and UUG). Among the codons with an RSCU value less than 1, except for AUA, CUA, and UGA, which terminate in A, the remaining codons conclude with either C or G. Due to the majority of genes having Ks values of 0, this leads to an ineffective Ka/Ks ratio (Figure 4C). Out of the 79 common genes, only 8 had valid values, with *ycf1* and *ndhD* exhibiting values greater than 1.

### 2.5. Phylogenetic Analysis

**Figure 5:**
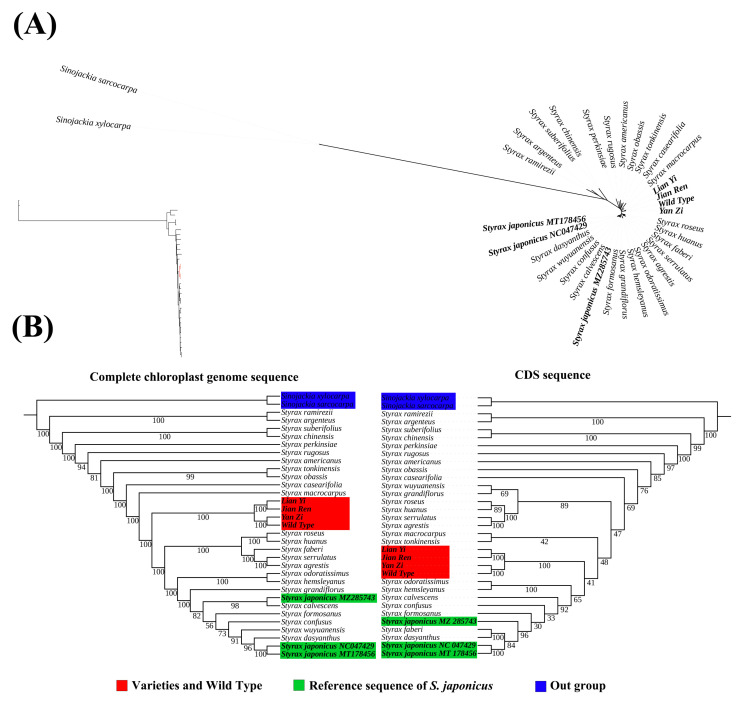
Phylogenetic tree analysis using the maximum likelihood (ML). (**A**) Phylogenetic topologies of 33 *Styrax* species; (**B**) phylogenetic tree constructed from complete chloroplast genome sequence (**left**) and CDS sequence (**right**).

The best-fit model GTR+G determined by ModelFinder was utilized for maximum likelihood analysis. Based on the *Styrax* topology, the interbranches within the genus are notably reduced, suggesting a lower level of divergence between *Styrax* species (Figure 5A). Phylogenetic analysis of the complete chloroplast genome and CDS sequences revealed that *S. japonicus* formed three clades within *Styrax*. The four samples sequenced in this study grouped together, with YZ and WT forming one branch, and LY and JR forming another. Three additional reference sequences reveal that *S. japonicus* (MZ285743) forms a branch with *S. calvescens* and as a sister clade to two other *S. japonicus* in the complete chloroplast genome tree (Figure 5B). However, in the CDS tree, *S. japonicus* (MZ285743) appears as a single branch, while the remaining two *S.japonicus* (NC047429 and MT178456) consistently form a branch in both trees. In general, the phylogenetic analysis revealed that seven samples of *S. japonicus* formed three distinct clusters, indicating a high level of diversity in the chloroplast genome and potential phylogenetic conflict within this species.

## 3. Discussion

In this study, we performed sequencing of three *S. japonicus* varieties and conducted a comparative analysis with the wild type and other *S. japonicus* to explore the correlation between leaf morphological variation and evolution in the chloroplast genome dimension. The results revealed a high degree of similarity among their chloroplast genomes; however, sliding window analysis identified potential mutation hotspots that could serve as DNA barcodes for the identification and germplasm resource of *S. japonicus* taxa. Additionally, (1) the boundary genes detection revealed varying degrees of expansion and contraction in the IR region of the three varieties; (2) *ndhD* and *ycf1* were found to be under positive selection; (3) phylogenetic analysis revealed the formation of three clades by seven *S. japonicus*. In the following, we will discuss and explore these three points.

### 3.1. IR Region Contraction within S. japonicus

The findings of this study demonstrate that the boundary genes in all *S. japonicus* are consistent; however, the IR regions of the three varieties exhibit varying degrees of contraction compared to different *S. japonicus* samples. Despite being minor, only a few bp, it is noteworthy that such changes occur within the same species.

The IR region of the chloroplast genome is generally considered to be highly conserved [36], but variations in its boundary genes can lead to changes in gene loss or the formation of pseudogenes [37]. This phenomenon is common in chloroplast genome evolution and is a major contributor to its length variation [38]. However, the expansion or contraction of IR regions can be attributed to various factors, including genome rearrangement [39], random mutations [40], evolutionary pressure [41], or gene transfer [42].

In conjunction with the current study, we hypothesize that the contraction of the IR region of *S. japonicus* in this study is primarily propelled by adaptive evolution due to specific environmental stress adaptation. This variation directly contributes to changes in genetic diversity, leading to the generation of new genetic variants. Moreover, it has been demonstrated that the expansion of the IR region can enhance the stability of the chloroplast genome [43]. Conversely, the contraction of the IR region in this study indicates a reduction in the structural stability of the chloroplast genome, which may be a primary factor contributing to phenotypic change among varieties.

### 3.2. Positive Selection of ndhD and ycf1

The results of the selection pressure analysis in this study revealed a positive selection of the *ndhD* and *ycf1* genes, directly indicating the adaptive evolution of the four *S. japonicus*. Positive selection may represent the optimization of gene function in response to environmental stress [44,45,46].

The *ndhD*-encoded protein is a constituent subunit of the chloroplast NDH complex [47], which plays a crucial role in cyclic electron transport and respiration around photosystem I (PSI), generating a transthylakoid membrane proton gradient and ATP for CO_2_ assimilation. The expression of the *ndhD* gene is subject to regulation through RNA editing. RNA editing can create start codons to control the efficiency of translation and promote gene expression. For instance, in the chloroplast genome of *Arabidopsis thaliana*, the ACG sequence is altered to AUG, thereby generating the start codon for *ndhD* [48]. Hirose and Sugiura also observed a correlation between the editing efficiency of tobacco *ndhD* and the stage of leaf development, with the highest efficiency occurring in the early stages and gradually decreasing [49]. Additionally, previous research has indicated that environmental factors such as temperature and light can impact the expression of the *ndhD* gene [50]. In this study, we speculate that four *S. japonicus ndhD* genes were positively selected, leading to alterations in the photosynthetic process and subsequent changes in the morphology of *S. japonicus*. Although research on this process is limited, these findings directly indicate the ongoing evolution of *S. japonicus*.

The *ycf1* gene is typically the largest in chloroplasts and is a component of the chloroplast inner membrane translocon [51]. However, this study found that *ycf1* was a pseudogene, a phenomenon also observed in rice, maize, palm, and some Geraniaceae species [52,53,54]. Additionally, the *ycf1* gene has been actively selected in other lineages, such as *Oenothera*, *Citrus*, and *Cardamine* [55,56,57]. If the genes encoded by chloroplasts become pseudogenes, it typically indicates their inability to encode functional proteins. In this study, *ycf1* was identified as a pseudogene due to the presence of a premature stop codon. However, we hypothesize that it may still possess certain functionalities, such as (1) influencing the expression of nearby genes due to the presence of enhancers or promoters in the sequence [58]; (2) transcribing into non-coding RNA with regulatory functions [59]; (3) becoming part of the transposable element, affecting the dynamic changes of the genome [60]. Of course, pseudogenes may also acquire new functions through evolutionary processes. The positive selection of *ycf1* as a pseudogene in this study provides robust support for the ongoing evolutionary event in the *S. japonicus* chloroplast genome, a phenomenon not previously observed in studies of this species.

In this section, we endeavor to speculate on the underlying factors contributing to chloroplast diversity in *S. japonicus* by conducting an analysis of *ndhD* and *ycf1*. Despite our limited understanding, these findings provide novel insights and establish a groundwork for future genetic manipulation and breeding initiatives.

### 3.3. Phylogenetic Analysis

The phylogenetic structure reconstructed based on the chloroplast genome in this study reveals conflicting relationships within seven *S. japonicus* samples. This discrepancy, not previously observed in *Styrax* studies, prompts a discussion of the reasons for these conflicts in the species’ phylogeny.

In previous studies, phylogenetic analysis based on chloroplast genomes revealed a close clustering of *S. japonicus* with *S. grandiflorus*, *S. confuses*, and *S. calvescens* [61], which is similar to the findings of the phylogenetic study of *Styrax* by Song et al. [62]. However, these analyses did not capture the full extent of chloroplast genome diversity in individual species. Our study, which combined analysis of published *S. japonicus* chloroplast genomes with four newly sequenced samples, revealed that *S. japonicus* forms at least three distinct clusters within *Styrax*; it shares sister branches with *S. calvescens*, *S. dasyanthus*, and *S. faberi*, respectively.

Generally, samples of the same species may diverge into different branches for several reasons. Firstly, genetic diversity can lead to significant variations within the species [63]. Secondly, geographic isolation or differences in flora distribution between populations may restrict gene flow [64]. Thirdly, niche differentiation can promote the development of various adaptive evolutionary paths within a species [65,66]. Fourth, mutations and gene recombination in sexual reproduction can introduce new genetic variations [67,68]. Additionally, population bottleneck events or founder effects of small populations can result in a reduction in genetic diversity, reflected in the phylogenetic tree where some samples may be markedly different from others [69,70,71]. Furthermore, hybridization and incomplete lineage sorting with closely related species may also contribute to the formation of distinct clades in phylogenetic analysis [16,17,72].

In combination with this study, we inferred that the broad distribution of *S. japonicus* and its overlapping habit with other *Styrax* species may increase the likelihood of hybridization and gene introgression, resulting in direct phylogenetic conflict and diversity of chloroplast genomes [73,74,75]. For example, Wang et al. conducted a comparison between domesticated soybean and wild-type soybean, revealing that gene introgression events contributed to the genetic diversity of domesticated soybean, as well as the asymmetric evolution of the nuclear genome and chloroplast genome [76].

Hybridization and gene introgression can result in recombination and mutation of the chloroplast genome, leading to the emergence of new chloroplast genotypes with potentially distinct biological functions and adaptations [77,78]. For instance, variations in chloroplast genes may impact photosynthesis efficiency, subsequently influencing plant growth and survival [79,80]. Gene introgression could also alter the selection pressure on certain genes within the chloroplast genome, thereby facilitating positive or negative selection processes and impacting the adaptive evolution of the chloroplast genome [81].

The complexity of relationships among the seven *S. japonicus* samples in this study indicates that the chloroplast genome of *S. japonicus* exhibits diversity and may be undergoing evolutionary divergence. Although the evolutionary mechanism has not been fully elucidated, the findings of this study provide compelling evidence for the evolution of the chloroplast genome of *S. japonicus* and establish a solid foundation for future germplasm resources.

## 4. Materials and Methods

### 4.1. Plant Materials

In this study, fresh blades of four samples were collected from the natural population of *S. japonicus* in Weide Mountain, Rongcheng, Shandong Province, China (122.422° E, 37.273° N).

**Figure 6:**
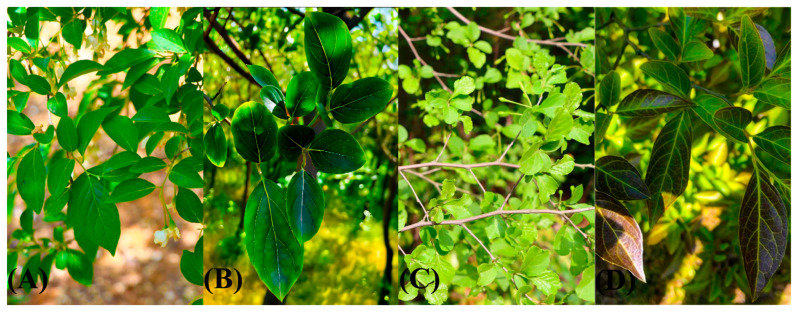
Leaf comparison of three *S.japonicus* varieties and wild type. (**A**): Wild type. (**B**): Jian Ren. (**C**): Lian Yi. (**D**): Yan Zi.

We designated them as Jian Ren (JR), Lian Yi (LY), Yan Zi (YZ), and the wild type (WT) for differentiation. To be specific, three varieties exhibited morphological variations in leaf color, shape, and texture when compared to the WT (Figure 6A). The leaves of JR are thick and leathery (Figure 6B), those of LY exhibit leaf folds (Figure 6C), and the leaves of YZ are purplish red in color (Figure 6D). The voucher specimens were deposited in the herbarium of Nanjing Forestry University.

The methods are exclusively conducted on *Styrax* plants for experimental purposes, strictly adhering to relevant institutional, national, and international guidelines and regulations.

### 4.2. DNA Extraction and Sequencing

The fresh and healthy leaf tissues of four samples were frozen in liquid nitrogen and stored in an Ultra-low temperature freezer for DNA extraction. The DNA extraction was carried out using the Plant Genomic DNA Kit (Nanjing Genepioneer Biotechnologies Inc., Nanjing, China) according to the manufacturer’s instructions. The concentration and purity of the DNA were evaluated using a Nandrop 2000 instrument (Thermo Fisher Scientific, Waltham, MA, USA). The nucleic acids utilized for sequencing demonstrated a purity range of 1.8–2.0, as indicated by A260/A280 ratios and sample concentrations ≥10 ng/μL. After the quality inspection of the genomic DNA was performed, the specific process included DNA sample detection, fragmentation, end repair, 3′ end plus A, adapter ligation, fragment selection by agarose gel electrophoresis, polymerase chain reaction (PCR) amplification for sequencing library formation, and quality verification. Subsequently, it underwent sequencing on the Illumina NovaSeq 6000 (Illumina, Cambridge, MA, USA) platform with a read length of 150 bp. The total DNA extracting and sequencing were conducted by Nanjing Genepioneer Biotechnologies Inc. (Nanjing, China).

### 4.3. Chloroplast Assembly and Annotation

Fastp v0.20.0 was used to trim the raw reads, and the high-quality clean data were obtained by removing the connector sequences and low-quality reads (the Filtering Criteria are in Appendix A). Bowtie2 v2.2.4 (http://bowtie-bio.sourceforge.net/bowtie2/index.shtml, accessed on 1 June 2024) [82] was used to align the clean data with the chloroplast genome database built by Genepioneer Biotechnologies in a very sensitive local mode. SPAdes v3.10.1 [83] https://microbialgenomicslab-spring2022.readthedocs.io/en/latest/GenomeAssemblies.html, accessed on 1 June 2024) was used to acquire seed sequences, and the contigs were obtained using the kmer iterative extend seed. The contig sequences were linked into scaffolds using SSPACE v2.0 [67,84] and then used in Gapfiller v2.1.1 [85] to fill the gaps [86] (the Assembly Process is in Appendix A). Two methods were used to annotate the chloroplast genomes to improve the accuracy of the annotation. First, protein-coding genes were annotated using Prodigal v2.6.3 (https://www.github.com/hyattpd/Prodigal, accessed on 1 June 2024). rRNA was predicted using Hmmer [87] (http://www.hmmer.org/, accessed on 1 June 2024), and tRNA was predicted using Aragorn v1.2.38 [88] (https://chlorobox.mpimp-golm.mpg.de/geseq.html, accessed on 1 June 2024). Second, the assembled sequences were compared using Blast v2.6 [89] (https://blast.ncbi.nlm.nih.gov/Blast.cgi, accessed on 1 June 2024) against related species published in the NCBI database. Subsequently, the two sets of annotations were compared and manually corrected. Finally, the chloroplast genome was mapped using the OGDRAW [90] (https://chlorobox.mpimp-golm.mpg.de/OGDraw.html, accessed on 1 June 2024). The chloroplast genome sequences were deposited in GenBank (GenBank accessions: PP853567-PP853569; OQ473820).

### 4.4. Chloroplast Genome Comparative Analysis

The boundaries of the plastome sequences collected and generated in this study were visualized in IRscope [91] (http://genocat.tools/tools/irscope.html, accessed on 1 June 2024). The Shufe-LAGAN model in the mVISTA program was utilized to compare the chloroplast genome structure of three varieties, with WT serving as a reference. DnaSP version 6 [92] was utilized to compute the nucleotide diversity (Pi) of coding sequences (CDS) and complete chloroplast genomes. The analysis employed a step size of 50 bp with a window length of 600 bp.

### 4.5. Repeat and Analysis of Codon Usage Bias

REPuter [93] (https://bibiserv.cebitec.uni-bielefeld.de/reputer, accessed on 1 June 2024) was employed to identify the dimensions and positions of forward, reverse, palindromic, and complementary repeats. Simple sequence repeats (SSRs) were determined using a Perl script MISA [94], encompassing mono-, di-, tri-, tetra-, penta-, and hexa-nucleotides with minimum thresholds of 10, 6, 5, 5, 5, and 5, respectively. For the analysis of codon bias, full-length CDS sequences were extracted for the three variants using ATG as the start codon and TGA, TAG, or TAA as the stop codon to determine codon usage preferences. CodonW was utilized to calculate the nucleotide composition at the third position (A3s, U3s, and G3s), codon adaptation index (CAI), codon bias index (CBI), and effective number of codons (ENC).

### 4.6. RSCU Analysis and Selective Pressures in the Evolution

Relative synonymous codon usage (RSCU) was analyzed using BioPython 1.84 [95]. The synonymous (Ks) and non-synonymous (Ka) substitution, as well as their ratios (Ka/Ks), were calculated by extracting and comparing the common coding sequences (CDS) of the four samples using Mafft 7.463 [96]. The YN algorithm was chosen in the KaKs calculator 3.0 [97] to consider the evolutionary characters of the sequence [14,98] for the purpose of elucidating the Ka/Ks value and conducting selection pressure analysis.

### 4.7. Phylogenetic Analysis

For the purpose of investigating the phylogenetic positions of the three *Styrax* varieties, a total of 31 *Styrax* chloroplast genomes were utilized for phylogenetic analysis, with two additional *Sinkjackia* species (*Sinojackia xylocarpa* and *Sinojackia sarcocarpa*) serving as outgroups.

In order to explore the phylogenetic relationships of *S. japonicus*, 31 *Styrax* contained 3 additional complete chloroplast genomes of *S. japonicus* (obtained from NCBI, accession number: MZ287543; NC047429; MT187456). Two datasets (complete chloroplast genomes and CDS sequences) were generated from chloroplast genomes, and after alignment, the optimal model was determined using ModelFinder [99]. Maximum-likelihood (ML) phylogenetic analysis was conducted using IQ-TREE 2.2.0 [100] with 1000 bootstrap replicates.

## 5. Conclusions

To explore the diversity of the chloroplast genome of *S. japonicus* and its effects on its morphology, we conducted the complete chloroplast genome sequencing of four samples from a natural population in Shandong, China, comprising three variants and one wild-type sample. Comparison with the previously published chloroplast genome of *S. japonicus* revealed a slight contraction in the IR region of four samples in this study, which may be indicative of adaptive evolution and indirectly suggests clues to genetic diversity. Both *ycf1* and *ndhD* underwent positive selection, with *ycf1* being identified as a pseudogene, indicating the adaptive evolution of *S. japonicus* to environmental stress. Phylogenetic analysis revealed that *S. japonicus* did not exhibit clustering or sister clades, suggesting that genetic diversity, geographic isolation, interbreeding, and other factors may have contributed to this phenomenon. In conclusion, a comparative analysis of the chloroplast genome suggests that *S. japonicus* may be undergoing divergent evolutionary paths, primarily driven by adaptive evolution. Furthermore, the observed variations in leaf morphology provide evidence for this phenomenon. While morphological changes are primarily regulated by the nuclear genome, the evolution of the chloroplast genome indirectly contributes to the development of these changes to some extent. Although the specific mechanisms underlying leaf deformation remain unknown, the diversity present in the chloroplast genome lays a foundation for future breeding and gene editing endeavors.

## Data Availability

The original contributions presented in this study are publicly available. These data can be found here: NCBI (GenBank accessions: PP853567-PP853569; OQ473820).

## Appendix A

**Filtering** **Criteria**

Fastp v0.20.0 (https://github.com/OpenGene/fastp, accessed on 1 June 2024) was used to filter the original data. The filtering criteria were as follows:

(1) Truncate the sequencing linker and primer sequence in the reads;
(2) Filter out the reads whose average quality value is <Q5;
(3) Filter out n reads whose number is >5;

The high-quality reads obtained after the above series of quality control steps were the clean data.

**Assembly** **Process**

SPAdes v3.10.1 [83] was used for genome assembly; kmer used 55, 87, and 121, respectively; and the assembly did not depend on the reference genome. However, due to the characteristics of second-generation sequencing, genomic repeats, genome-specific structure, and other reasons, the complete circular genome sequence could not be directly obtained by one-time splicing. Other strategies were used to obtain the complete circular genome sequence. The assembly process was divided into the following seven steps:

Step 1: The seed sequence of the chloroplast genome was obtained by assembling the cpDNA sequences with SPAdes v3.10.1.

Step 2: kmer iterative extend seed if the result of step 2 was a contig, the result was determined to be a pseudogenome sequence, and step 6 was performed directly.

Step 3: Connect the contig sequence obtained in step 2 with SSPACE v2.0 [84] to obtain the scaffolds.

Step 4: Use Gapfiller v2.1.1 [85] to make up the gaps for the scaffold sequences obtained in step 3.

Step 5: If a gap still exists after these operations, design primers, conduct PCR sequencing, and reassemble until the complete pseudogenome sequence is obtained.

Step 6: The sequencing reads were aligned to the pseudogenome to correct the genome.

Step 7: According to the structure of the chloroplast, the corrected pseudogenome was rearranged to obtain the complete chloroplast circular genome sequence.

## Appendix B

**Table A1:** Characteristics of assembled chloroplast genome basic structure.

Species	Total Size	GC Content (%)	LSC Size	IR Size	SSC Size	Protein Coding Genes	rRNA	tRNA	NCBI Accession Number
Lian Yi	157,962 bp	36.96%	87,637 bp	26,040 bp	18,245 bp	87	8	37	PP853569
Jian Ren	157,962 bp	36.96%	87,637 bp	26,040 bp	18,245 bp	87	8	37	PP853567
Yan Zi	157,914 bp	36.98%	87,549 bp	26,048 bp	18,269 bp	87	8	37	PP853568
Wild type	157,914 bp	36.97%	87,549 bp	26,048 bp	18,269 bp	87	8	37	OQ473820

**Table A2:** Lists of genomic genes for Jian Ren, Lian Yi, Yan Zi, and wild type. Notes: Gene*: Gene with one introns; Gene**: Gene with two introns; #Gene: Pseudogene; Gene(2): Number of copies of multi-copy genes.

Category	Function	Jian Ren	Lian Yi	Yan Zi	Wild Type
Photosynthesis	Subunits of photosystem I	*psaA*, *psaB*, *psaC*, *psaI*, *psaJ*			
	Subunits of photosystem II	*psbA*, *psbB*, *psbC*, *psbD*, *psbE*, *psbF*, *psbH*, *psbI*, *psbJ*, *psbK*, *psbL*, *psbM*, *psbN*, *psbT*			
	Subunits of NADH dehydrogenase	*ndhA**, *ndhB*(2)*, *ndhC*, *ndhD*, *ndhE*, *ndhF*, *ndhG*, *ndhH*, *ndhI*, *ndhJ*, *ndhK*			
	Subunits of cytochrome b/f complex	*petA*, *petB**, *petD**, *petG*, *petL*, *petN*			
	Subunits of ATP synthase	*atpA*, *atpB*, *atpE*, *atpF**, *atpH*, *atpI*			
	Large subunit of rubisco	*rbcL*			
	Subunits photochlorophyllide reductase	-			
Self-replication	Proteins of large ribosomal subunit	*rpl14*, *rpl16**, *rpl2*(2)*, *rpl20*, *rpl22*, *rpl23(2)*, *rpl32*, *rpl33*, *rpl36*			
	Proteins of small ribosomal subunit	*rps11*, *rps12**(2)*, *rps14*, *rps15*, *rps16**, *rps18*, *rps19*, *rps2*, *rps3*, *rps4*, *rps7(2)*, *rps8*			
	Subunits of RNA polymerase	*rpoA*, *rpoB*, *rpoC1**, *rpoC2*			
	Ribosomal RNAs	*rrn16(2)*, *rrn23(2)*, *rrn4.5(2)*, *rrn5(2)*			
	Transfer RNAs	*trnA-UGC*(2)*, *trnC-GCA*, *trnD-GUC*, *trnE-UUC*, *trnF-GAA*, *trnG-GCC**, *trnG-UCC*, *trnH-GUG*, *trnI-CAU(2)*, *trnI-GAU*(2)*, *trnK-UUU**, *trnL-CAA(2)*, *trnL-UAA**, *trnL-UAG*, *trnM-CAU*, *trnN-GUU(2)*, *trnP-UGG*, *trnQ-UUG*, *trnR-ACG(2)*, *trnR-UCU*, *trnS-GCU*, *trnS-GGA*, *trnS-UGA*, *trnT-GGU*, *trnT-UGU*, *trnV-GAC(2)*, *trnV-UAC**, *trnW-CCA*, *trnY-GUA*, *trnfM-CAU*			
Other genes	Maturase	*matK*			
	Protease	*clpP***			
	Envelope membrane protein	*cemA*			
	Acetyl–CoA carboxylase	*accD*			
	c-type cytochrome synthesis gene	*ccsA*			
	Translation initiation factor	*infA*			
	other	-			
Genes of unknown function	Conserved hypothetical chloroplast ORF	*#ycf1*, *lhbA*, *ycf1*, *ycf15(2)*, *ycf2(2)*, *ycf3***, *ycf4*			

## References

[1] Prabhudas S.K., Prayaga S., Madasamy P., Natarajan P. (2016). Shallow Whole Genome Sequencing for the Assembly of Complete Chloroplast Genome Sequence of *Arachis hypogaea* L. Front. Plant Sci..

[2] Douglas S.E. (1998). Plastid Evolution: Origins, Diversity, Trends. Curr. Opin. Genet. Dev..

[3] Shinozaki K., Ohme M., Tanaka M., Wakasugi T., Hayashida N., Matsubayashi T., Zaita N., Chunwongse J., Obokata J., Yamaguchi-Shinozaki K. (1986). The Complete Nucleotide Sequence of the Tobacco Chloroplast Genome: Its Gene Organization and Expression. EMBO J..

[4] Sassenrath-Cole G.F. (1999). Photosynthesis, a Comprehensive Treatise. Crop Sci..

[5] Alkatib S., Fleischmann T.T., Scharff L.B., Bock R. (2012). Evolutionary Constraints on the Plastid Trna Set Decoding Methionine and Isoleucine. Nucleic Acids Res..

[6] Rogalski M., Karcher D., Bock R. (2008). Superwobbling Facilitates Translation with Reduced Trna Sets. Nat. Struct. Mol. Biol..

[7] Dong W.P., Xu C., Wen J., Zhou S.L. (2020). Evolutionary Directions of Single Nucleotide Substitutions and Structural Mutations in the Chloroplast Genomes of the Family Calycanthaceae. BMC Evol. Biol..

[8] Zhang J., Wang Y., Chen T., Chen Q., Wang L., Liu Z.S., Wang H., Xie R., He W., Li M. (2021). Evolution of Rosaceae Plastomes Highlights Unique *Cerasus* Diversification and Independent Origins of Fruiting Cherry. Front. Plant Sci..

[9] Cao Z.Y., Yang L.Y., Xin Y.X., Xu W.B., Li Q.S., Zhang H.R., Tu Y.X., Song Y., Xin P.Y. (2023). Comparative and Phylogenetic Analysis of Complete Chloroplast Genomes from Seven *Neocinnamomum* Taxa (Lauraceae). Front. Plant Sci..

[10] Shi W.B., Song W.C., Chen Z.M., Cai H.H., Gong Q., Liu J., Shi C., Wang S. (2023). Comparative Chloroplast Genome Analyses of Diverse *Phoebe* (Lauraceae) Species Endemic to China Provide Insight into Their Phylogeographical Origin. PeerJ.

[11] Dong W.P., Xu C., Cheng T., Lin K., Zhou S.L. (2013). Sequencing Angiosperm Plastid Genomes Made Easy: A Complete Set of Universal Primers and a Case Study on the Phylogeny of Saxifragales. Genome Biol. Evol..

[12] Wu Z.Q., Ge S. (2012). The Phylogeny of the Bep Clade in Grasses Revisited: Evidence from the Whole-Genome Sequences of Chloroplasts. Mol. Phylogenet. Evol..

[13] Li H.T., Yi T.S., Gao L.M., Ma P.F., Zhang T., Yang J.B., Gitzendanner M.A., Fritsch P.W., Cai J., Luo Y. (2019). Origin of Angiosperms and the Puzzle of the Jurassic Gap. Nat. Plants.

[14] Zhang R., Wang Y.H., Jin J.J., Stull G.W., Bruneau A., Cardoso D., De Queiroz L.P., Moore M.J., Zhang S.D., Chen S.Y. (2020). Exploration of Plastid Phylogenomic Conflict Yields New Insights into the Deep Relationships of Leguminosae. Syst. Biol..

[15] Corriveau J.L., Coleman A.W. (1988). Rapid Screening Method to Detect Potential Biparental Inheritance of Plastid DNA and Results for over 200 Angiosperm Species. Am. J. Bot..

[16] Joly S., McLenachan P.A., Lockhart P.J. (2009). A Statistical Approach for Distinguishing Hybridization and Incomplete Lineage Sorting. Am. Nat..

[17] Petit R.J., Excoffier L. (2009). Gene Flow and Species Delimitation. Trends Ecol. Evol..

[18] Song Y., Yu W.-B., Tan Y.H., Liu B., Yao X., Jin J.J., Padmanaba M., Yang J.B., Corlett R.T. (2017). Evolutionary Comparisons of the Chloroplast Genome in Lauraceae and Insights into Loss Events in the Magnoliids. Genome Biol. Evol..

[19] Chen J.F., Yu R.X., Dai J.H., Liu Y., Zhou R.C. (2020). The Loss of Photosynthesis Pathway and Genomic Locations of the Lost Plastid Genes in a Holoparasitic Plant *Aeginetia indica*. BMC Plant Biol..

[20] Wu Z.H., Liao R., Yang T.G., Dong X., Lan D.Q., Qin R., Liu H. (2020). Analysis of Six Chloroplast Genomes Provides Insight into the Evolution of *Chrysosplenium* (Saxifragaceae). BMC Genom..

[21] Park K.R., Kim S., Cho M., Kang S.W., Yun H.M. (2020). Effects of Pin on Osteoblast Differentiation and Matrix Mineralization through Runt-Related Transcription Factor. Int. J. Mol. Sci..

[22] Horimoto T., Koshioka M., Kubota S., Mander L.N., Hirai N., Ishida N., Suh J.K., Lee A.K., Roh M.S. (2011). Effect of Warm and Cold Stratification on 1h-Nmr Profiles, Endogenous Gibberellins and Abscisic Acid in *Styrax japonicus* Seeds. Hortic. Environ. Biotechnol..

[23] Chen C., Chen H., Ni M., Yu F.Y. (2021). A Study on Petal Morphological and Physiological Characteristics of *Styrax japonicus* During the Flowering Period. Agronomy.

[24] Kitagawa I., Imaukura Y., Hayashi T., Yosioka I. (1974). Structure of Desacyl-Jegosaponin, a Common Desacyl Derivative of Jegosaponin Isolated from Pericarps of *Styrax japonica* Sieb. Et Zucc. Chem. Pharm. Bull..

[25] Min B.S., Na M.K., Oh S.R., Ahn K.S., Jeong G.S., Li G., Lee S.K., Joung H., Lee H.K. (2004). New Furofuran and Butyrolactone Lignans with Antioxidant Activity from the Stem Bark of *Styrax japonica*. J. Nat. Prod..

[26] He L., Zhou Y., Wan G., Wang W., Zhang N., Yao L. (2022). Antinociceptive Effects of Flower Extracts and the Active Fraction from *Styrax japonicus*. J. Ethnopharmacol..

[27] Ren J., Fang A., Jiao S., Li R., Huang Y., Ni X., Zhang Y., Ma Y., Li S., Li J. (2024). Lignans from the Leaves of *Styrax japonicus* and Their Anti-Inflammatory Activity. Fitoterapia.

[28] Li W., Zhang C.P., Jiang X.Q., Liu Q., Liu Q.H., Wang K.L. (2018). De Novo Transcriptomic Analysis and Development of Est–Ssrs for *Styrax japonicus*. Forests.

[29] Fritsch P.W. (1999). Phylogeny of Styrax Based on Morphological Characters, with Implications for Biogeography and Infrageneric Classification. Syst. Bot..

[30] Huang S.M. (1994). Systematic Position and Geographical Distribution of Styracaceae. J. Trop. Subtrop. Bot..

[31] Huang Y.L., Fritsch P., Shi S.H. (2003). A Revision of the Imbricate Group of *Styrax* Series *Cyrta* (Styracaceae) in Asia. Ann. Mo. Bot. Gard..

[32] Ju Y.Q., Zhang C.P., Li W., Qian C., Qu Y.M., Zou Z.X., Zhao H., Li L.L. (2023). Variation in the Calyx Color in Two *Styrax japonicus* Varieties Is Attributed to Varied Anthocyanin Levels as Revealed by Integrated Metabolomic and Transcriptomic Analyses. Forests.

[33] Lee S.H., Kim J., Park H.S., Koo H., Waminal N.E., Pellerin R.J., Shim H., Lee H.O., Kim E., Park J.Y. (2022). Genome Structure and Diversity among *Cynanchum wilfordii* Accessions. BMC Plant Biol..

[34] Tian X.Y., Guo J., Song Y., Yu Q.F., Liu C., Fu Z.X., Shi Y.H., Shao Y.Z., Yuan Z.L. (2024). Intraspecific Differentiation of *Lindera obtusiloba* as Revealed by Comparative Plastomic and Evolutionary Analyses. Ecol. Evol..

[35] Sarwar G., Anwar T., Chaudhary M.S., Jamil M., Kamal A., Mustafa A.M.A., Al-Ghamdi A.A., Ullah F., Zaman W. (2023). Study of Comparative Morphology of Eight Cultivated Genotypes of *Olea europaea* L. Horticulturae.

[36] Zhu A., Guo W., Gupta S., Fan W., Mower J.P. (2016). Evolutionary Dynamics of the Plastid Inverted Repeat: The Effects of Expansion, Contraction, and Loss on Substitution Rates. New Phytol..

[37] Guo Y.Y., Yang J.X., Bai M.Z., Zhang G.Q., Liu Z.J. (2021). The Chloroplast Genome Evolution of Venus Slipper (*Paphiopedilum*): Ir Expansion, Ssc Contraction, and Highly Rearranged SSC Regions. BMC Plant Biol..

[38] Guo S., Guo L., Zhao W., Xu J., Li Y., Zhang X., Shen X., Wu M., Hou X. (2018). Complete Chloroplast Genome Sequence and Phylogenetic Analysis of *Paeonia ostii*. Molecules.

[39] Zhang X.F., Landis J.B., Wang H.X., Zhu Z.X., Wang H.F. (2021). Comparative Analysis of Chloroplast Genome Structure and Molecular Dating in Myrtales. BMC Plant Biol..

[40] Lynch M., Koskella B., Schaack S. (2006). Mutation Pressure and the Evolution of Organelle Genomic Architecture. Science.

[41] Bungard R.A. (2004). Photosynthetic Evolution in Parasitic Plants: Insight from the Chloroplast Genome. Bioessays.

[42] Xiong A.S., Peng R.H., Zhuang J., Gao F., Zhu B., Fu X.Y., Xue Y., Jin X.F., Tian Y.S., Zhao W. (2009). Gene Duplication, Transfer, and Evolution in the Chloroplast Genome. Biotechnol. Adv..

[43] Sun M., Zhang J., Huang T., Yang M., Ma L., Duan L. (2022). Genome Structure and Variation of *Reynoutria japonica Houtt*. Chloroplast Genome. Sheng Wu Gong Cheng Xue Bao.

[44] Hasegawa M., Zhong B., Zhong Y. (2009). Adaptive Evolution of Chloroplast Genomes in Ancestral Grasses. Plant Signal Behav..

[45] Wang Y., Wen F., Hong X., Li Z., Mi Y., Zhao B. (2022). Comparative Chloroplast Genome Analyses of *Paraboea* (Gesneriaceae): Insights into Adaptive Evolution and Phylogenetic Analysis. Front. Plant Sci..

[46] Yang Q., Fu G.F., Wu Z.Q., Li L., Zhao J.L., Li Q.J. (2021). Chloroplast Genome Evolution in Four Montane Zingiberaceae Taxa in China. Front. Plant Sci..

[47] Li Q.H., He Z.H., Mi H.L. (2013). The Research Progress of Chloroplast Nad(P)H Dehydrogenase (Ndh) Complex. Zhiwu Shengli Xuebao/Plant Physiol. J..

[48] Kotera E., Tasaka M., Shikanai T. (2005). A Pentatricopeptide Repeat Protein Is Essential for Rna Editing in Chloroplasts. Nature.

[49] Hirose T., Sugiura M. (1997). Both Rna Editing and Rna Cleavage Are Required for Translation of Tobacco Chloroplast Ndhd Mrna: A Possible Regulatory Mechanism for the Expression of a Chloroplast Operon Consisting of Functionally Unrelated Genes. EMBO J..

[50] Karcher D., Bock R. (2002). The Amino Acid Sequence of a Plastid Protein Is Developmentally Regulated by Rna Editing. J. Biol. Chem..

[51] Kikuchi S., Bédard J., Hirano M., Hirabayashi Y., Oishi M., Imai M., Takase M., Ide T., Nakai M. (2013). Uncovering the Protein Translocon at the Chloroplast Inner Envelope Membrane. Science.

[52] Maier R.M., Neckermann K., Igloi G.L., Kössel H. (1995). Complete Sequence of the Maize Chloroplast Genome: Gene Content, Hotspots of Divergence and Fine Tuning of Genetic Information by Transcript Editing. J. Mol. Biol..

[53] Weng M.L., Blazier J.C., Govindu M., Jansen R.K. (2014). Reconstruction of the Ancestral Plastid Genome in Geraniaceae Reveals a Correlation between Genome Rearrangements, Repeats, and Nucleotide Substitution Rates. Mol. Biol. Evol..

[54] Yang M., Zhang X., Liu G., Yin Y., Chen K., Yun Q., Zhao D., Al-Mssallem I.S., Yu J. (2010). The Complete Chloroplast Genome Sequence of Date Palm (*Phoenix dactylifera* L.). PLoS ONE.

[55] Carbonell-Caballero J., Alonso R., Ibañez V., Terol J., Talon M., Dopazo J. (2015). A Phylogenetic Analysis of 34 Chloroplast Genomes Elucidates the Relationships between Wild and Domestic Species within the Genus Citrus. Mol. Biol. Evol..

[56] Greiner S., Wang X., Herrmann R.G., Rauwolf U., Mayer K., Haberer G., Meurer J. (2008). The Complete Nucleotide Sequences of the 5 Genetically Distinct Plastid Genomes of Oenothera, Subsection *Oenothera*: II. A Microevolutionary View Using Bioinformatics and Formal Genetic Data. Mol. Biol. Evol..

[57] Hu S., Sablok G., Wang B., Qu D., Barbaro E., Viola R., Li M., Varotto C. (2015). Plastome Organization and Evolution of Chloroplast Genes in *Cardamine* Species Adapted to Contrasting Habitats. BMC Genom..

[58] Xie J.B., Chen S.S., Xu W.J., Zhao Y.Y., Zhang D.Q. (2019). Origination and Function of Plant Pseudogenes. Plant Signal. Behav..

[59] Zhang J.M., Pontoppidan B., Xue J.P., Rask L., Meijer J. (2002). The Third Myrosinase Gene Tgg3 in *Arabidopsis thaliana* Is a Pseudogene Specifically Expressed in Stamen and Petal. Physiol. Plant..

[60] Yadav S., Kalwan G., Meena S., Gill S.S., Yadava Y.K., Gaikwad K., Jain P.K. (2023). Unravelling the Due Importance of Pseudogenes and Their Resurrection in Plants. Plant Physiol. Biochem..

[61] Tong T.T., Shao L.L., Peng Z.H. (2020). The Complete Chloroplast Genome of *Styrax japonicus* (Styracaceae), a Deciduous Tree Distributed in East Asia. Mitochondrial DNA Part B Resour..

[62] Song Y., Zhao W.J., Xu J., Li M.F., Zhang Y.J. (2022). Chloroplast Genome Evolution and Species Identification of *Styrax* (Styracaceae). Biomed. Res. Int..

[63] Daniell H., Lin C.S., Yu M., Chang W.J. (2016). Chloroplast Genomes: Diversity, Evolution, and Applications in Genetic Engineering. Genome Biol..

[64] Lo E.Y., Stefanović S., Dickinson T.A. (2009). Population Genetic Structure of Diploid Sexual and Polyploid Apomictic Hawthorns (*Crataegus*; Rosaceae) in the Pacific Northwest. Mol. Ecol..

[65] Moen D., Morlon H. (2014). Why Does Diversification Slow Down?. Trends Ecol. Evol..

[66] Rabosky D.L. (2009). Ecological Limits and Diversification Rate: Alternative Paradigms to Explain the Variation in Species Richness among Clades and Regions. Ecol. Lett..

[67] Bernstein H., Byerly H.C., Hopf F.A., Michod R.E. (1985). Genetic Damage, Mutation, and the Evolution of Sex. Science.

[68] Charlesworth B. (1990). Mutation-Selection Balance and the Evolutionary Advantage of Sex and Recombination. Genet. Res..

[69] Alsos I.G., Ehrich D., Eidesen P.B., Solstad H., Westergaard K.B., Schönswetter P., Tribsch A., Birkeland S., Elven R., Brochmann C. (2015). Long-Distance Plant Dispersal to North Atlantic Islands: Colonization Routes and Founder Effect. AoB Plants.

[70] Zhang R., Guo Z., Fang L., Zhong C., Duke N.C., Shi S. (2022). Population Subdivision Promoted by a Sea-Level-Change-Driven Bottleneck: A Glimpse from the Evolutionary History of the Mangrove Plant *Aegiceras corniculatum*. Mol. Ecol..

[71] Major T., Gindele R., Balogh G., Bárdossy P., Bereczky Z. (2021). Founder Effects in Hereditary Hemorrhagic Telangiectasia. J. Clin. Med..

[72] Yang L., Su D., Chang X., Foster C.S.P., Sun L., Huang C.H., Zhou X., Zeng L., Ma H., Zhong B. (2020). Phylogenomic Insights into Deep Phylogeny of Angiosperms Based on Broad Nuclear Gene Sampling. Plant Commun..

[73] Wang G., Zhang X., Herre E.A., McKey D., Machado C.A., Yu W.B., Cannon C.H., Arnold M.L., Pereira R.A.S., Ming R. (2021). Genomic Evidence of Prevalent Hybridization Throughout the Evolutionary History of the Fig-Wasp Pollination Mutualism. Nat. Commun..

[74] Li X., Wei G., El-Kassaby Y.A., Fang Y. (2021). Hybridization and Introgression in Sympatric and Allopatric Populations of Four Oak Species. BMC Plant Biol..

[75] Vargas O.M., Ortiz E.M., Simpson B.B. (2017). Conflicting Phylogenomic Signals Reveal a Pattern of Reticulate Evolution in a Recent High-Andean Diversification (Asteraceae: Astereae: *Diplostephium*). New Phytol..

[76] Wang X., Chen L., Ma J. (2019). Genomic Introgression through Interspecific Hybridization Counteracts Genetic Bottleneck During Soybean Domestication. Genome Biol..

[77] Kurata S., Sakaguchi S., Kurashima O., Ogawa R., Suyama Y., Nishida S., Ito M. (2024). Refugia within Refugium of *Geranium yesoense* Varieties: A Follow-up Study Using Chloroplast Genome Sequencing Data of Specimens from Mt. Asama, Japan. Biol. J. Linn. Soc..

[78] Lange C.B.A., Hauser T.P., Deichmann V., Orgaard M. (2022). Hybridization and Complex Evolution of *Barbarea vulgaris* and Related Species (Brassicaceae). Mol. Phylogenet. Evol..

[79] You J.L., Lougheed S.C., Zhao Y., Zhang G.J., Liu W.S., Lu F., Wang Y.G., Zhang W.J., Yang J., Qiong L. (2022). Comparative Phylogeography Study Reveals Introgression and Incomplete Lineage Sorting During Rapid Diversification of Rhodiola. Ann. Bot..

[80] Zhang H.X., Zhang X.F., Zhang J. (2024). Genetic Divergence and Evolutionary Adaption of Four Wild Almond Species (*Prunus* Spp. L.). Forests.

[81] Stubbs R.L., Theodoridis S., Mora-Carrera E., Keller B., Potente G., Yousefi N., Jay P., Léveillé-Bourret É., Choudhury R.R., Celep F. (2024). The Genomes of Darwin’s Primroses Reveal Chromosome-Scale Adaptive Introgression and Differential Permeability of Species Boundaries. New Phytol..

[82] Langmead B., Salzberg S.L. (2012). Fast Gapped-Read Alignment with Bowtie 2. Nat. Methods.

[83] Bankevich A., Nurk S., Antipov D., Gurevich A.A., Dvorkin M., Kulikov A.S., Lesin V.M., Nikolenko S.I., Pham S., Prjibelski A.D. (2012). Spades: A New Genome Assembly Algorithm and Its Applications to Single-Cell Sequencing. J. Comput. Biol..

[84] Boetzer M., Pirovano W. (2014). Sspace-Longread: Scaffolding Bacterial Draft Genomes Using Long Read Sequence Information. BMC Bioinform..

[85] Boetzer M., Pirovano W. (2012). Toward Almost Closed Genomes with Gapfiller. Genome Biol..

[86] Xiong Y., Xiong Y., He J., Yu Q., Zhao J., Lei X., Dong Z., Yang J., Peng Y., Zhang X. (2020). The Complete Chloroplast Genome of Two Important Annual Clover Species, *Trifolium alexandrinum* and *T. resupinatum*: Genome Structure, Comparative Analyses and Phylogenetic Relationships with Relatives in Leguminosae. Plants.

[87] Potter S.C., Luciani A., Eddy S.R., Park Y., Lopez R., Finn R.D. (2018). Hmmer Web Server: 2018 Update. Nucleic Acids Res..

[88] Laslett D., Canback B. (2004). Aragorn, a Program to Detect Trna Genes and Tmrna Genes in Nucleotide Sequences. Nucleic Acids Res..

[89] McGinnis S., Madden T.L. (2004). Blast: At the Core of a Powerful and Diverse Set of Sequence Analysis Tools. Nucleic Acids Res..

[90] Greiner S., Lehwark P., Bock R. (2019). Organellargenomedraw (Ogdraw) Version 1.3.1: Expanded Toolkit for the Graphical Visualization of Organellar Genomes. Nucleic Acids Res..

[91] Amiryousefi A., Hyvönen J., Poczai P. (2018). Irscope: An Online Program to Visualize the Junction Sites of Chloroplast Genomes. Bioinformatics.

[92] Rozas J., Ferrer-Mata A., Sánchez-DelBarrio J.C., Guirao-Rico S., Librado P., Ramos-Onsins S.E., Sánchez-Gracia A. (2017). Dnasp 6: DNA Sequence Polymorphism Analysis of Large Data Sets. Mol. Biol. Evol..

[93] Kurtz S., Choudhuri J.V., Ohlebusch E., Schleiermacher C., Stoye J., Giegerich R. (2001). Reputer: The Manifold Applications of Repeat Analysis on a Genomic Scale. Nucleic Acids Res..

[94] Beier S., Thiel T., Münch T., Scholz U., Mascher M. (2017). Misa-Web: A Web Server for Microsatellite Prediction. Bioinformatics.

[95] Cock P.J., Antao T., Chang J.T., Chapman B.A., Cox C.J., Dalke A., Friedberg I., Hamelryck T., Kauff F., Wilczynski B. (2009). Biopython: Freely Available Python Tools for Computational Molecular Biology and Bioinformatics. Bioinformatics.

[96] Katoh K., Standley D.M. (2013). Mafft Multiple Sequence Alignment Software Version 7: Improvements in Performance and Usability. Mol. Biol. Evol..

[97] Zhang Z. (2022). Kaks_Calculator 3.0: Calculating Selective Pressure on Coding and Non-Coding Sequences. Genom. Proteom. Bioinform..

[98] Zeng S., Zhou T., Han K., Yang Y., Zhao J., Liu Z.L. (2017). The Complete Chloroplast Genome Sequences of Six Rehmannia Species. Genes.

[99] Kalyaanamoorthy S., Minh B.Q., Wong T.K.F., von Haeseler A., Jermiin L.S. (2017). Modelfinder: Fast Model Selection for Accurate Phylogenetic Estimates. Nat. Methods.

[100] Nguyen L.T., Schmidt H.A., von Haeseler A., Minh B.Q. (2015). Iq-Tree: A Fast and Effective Stochastic Algorithm for Estimating Maximum-Likelihood Phylogenies. Mol. Biol. Evol..

